# Persistent Social Inequality in Adolescent Health Indicators 1991–2022: Trend Study From Denmark

**DOI:** 10.3389/ijph.2024.1607698

**Published:** 2024-11-28

**Authors:** Bjørn E. Holstein, Mogens Trab Damsgaard, Trine Pagh Pedersen, Mette Rasmussen, Julie Ellegaard Ibáñez Román, Mette Toftager, Katrine Rich Madsen

**Affiliations:** ^1^ National Institute of Public Health, University of Southern Denmark, Copenhagen, Denmark; ^2^ Department of Sports Science and Clinical Biomechanics, University of Southern Denmark, Odense, Denmark

**Keywords:** adolescence, HBSC, physical health, mental health, health behaviour, social inequality, socioeconomic inequality, trend study

## Abstract

**Objectives:**

To examine trends in socioeconomic inequality in adolescent health over three decades, across fifteen health indicators: overweight, underweight, headache, stomachache, backpain, emotional symptoms, difficulties falling asleep, loneliness, low life satisfaction, low self-rated heath, smoking, drunkenness, physical inactivity, low vegetable intake, and inadequate toothbrushing.

**Methods:**

The Health Behaviour in School-aged Children (HBSC) study in Denmark included nine identical surveys of 11–15-year-olds from 1991 to 2022, n = 35,423. For each health indicator, we measured absolute and relative socioeconomic inequality by prevalence differences and odds ratios between low and high socioeconomic groups.

**Results:**

There was socioeconomic inequality in thirteen health indicators, e.g., the OR (95% CI) for overweight in low vs. high socioeconomic groups was 2.22 (1.95–2.49). This social inequality persisted across health indicators throughout the study period with two deviations: Underweight was not associated with socioeconomic background and drunkenness was persistently most prevalent in higher socioeconomic groups.

**Conclusion:**

The political efforts to reduce socioeconomic inequality in health seems to have failed. It is important to improve monitoring of adolescent health and implement improved policies to tackle socioeconomic inequality in adolescent health.

## Introduction

Social inequality in health refers to systematic differences in health between socioeconomic groups. There is ample evidence that adolescents from lower socioeconomic groups have higher risk of poor health and harmful health behaviours [1, 2]. Social inequality in child and adolescent health is a public health concern because the full health potential of children and adolescents from lower socioeconomic groups are not fulfilled [2, 3]. In many countries there has been a strong political interest in reducing social inequalities in health in the past decades [4]. This paper addresses time trends in social inequality in adolescent health from 1991 to 2022 in a country with a strong political desire to reduce this inequality. The analyses include 15 indicators of physical health, mental health, and health behaviours to provide a comprehensive picture of social inequality in adolescent health.

Most indicators of poor physical and mental health show higher prevalence with lower socioeconomic status among adolescents. This is the case for overweight [5–8], pain [9–12], psychological distress [12–16], difficulties falling asleep [17], loneliness [18, 19], poor life satisfaction [20] and poor self-rated health [21, 22]. Adolescents in lower socioeconomic strata have higher prevalences of unhealthy behaviours [23] including smoking [24–26], low vegetable intake [6, 27], physical inactivity [6], and infrequent toothbrushing [28]. Two exceptions are observed. One is underweight, which is not associated with socioeconomic status in high-income countries [29–31]. The other is binge drinking or drunkenness, where the association with socioeconomic status varies across countries [32, 33].

Although social inequality in health is well documented, less is known about secular trends in social inequality in adolescent health. In many domains of adolescent health, the social inequality in health has been either widening or persistent during the past decades. Elgar et al. studied trends from 2002 to 2010 and found widening social inequality in several health indicators, but not life satisfaction [34]. Lampert et al. analysed trends from 2003 to 2017 in general health, mental health, physical activity, consumption of sugary soft drinks, and smoking [35]. They found persistent and, in some cases, widening health inequalities, although sometimes with different developments in absolute and relative social inequality. Moor et al. found a stable pattern of social inequality in life satisfaction, self-rated health, fruit and vegetable consumption, and physical activity from 2009 to 2022 in Germany [36]. Hammani et al. found widening social inequalities from 2002 to 2018 in overweight, physical symptoms, low life satisfaction, and poor self-rated health among Canadian adolescents [37]. Studies of trends in social inequality in weight status among adolescents show widening social inequality in overweight and obesity in the past decades [5, 6, 31, 38]. Social inequalities in underweight has not changed much in Western countries between the 1990s and 2018 [29]. There are few studies of trends in social inequality in pain, psychological distress, loneliness, difficulties in falling asleep, and poor self-rated health. The findings vary by country, but most of the studies covering parts of the period 1990–2018 found persistent or slightly increasing social inequality [1, 10, 13, 39]. Two exceptions show diminishing social inequality due to an increasing rate of health problems among students from higher socioeconomic strata: A study by Madsen et al. showed diminishing social inequality in loneliness 1991–2014 [18] and a study by Due et al. showed diminishing social inequality in emotional symptoms 1991–2014 [40].

Studies analysing trends in social inequality in adolescent smoking since 1990 showed mixed results. Findings indicate a widening gap [25, 41], persistent social inequality [42], a diminishing gap [35] or differening trends for absolute and relative social inequality [24]. Few studies address changes in social inequality for other health behaviours since 1990 and most of these studies report persistent social inequality, however often with differences across countries, sex- and age groups [6, 27, 43–46].

Most studies on trends in social inequality in adolescent health focus on one or few indicators of adolescent health and cover relatively short periods. There is a need for studies which cover a broad range of health indicators over extended periods. Such studies can give insights into the full picture of inequalities of adolescent health and may help determine the success of political efforts to reduce health inequality. Therefore, the objective of this study was to analyse secular trends in social inequality across numerous indicators of adolescent health over a 31-year period, from 1991 to 2022. The study focused on adolescents in Denmark, a country with high *per capita* income, a high human development index, a relatively low income inequality, a strong political desire to reduce social inequality in health, and a comprehensive tax-financed welfare system.

There are several studies on changes in social inequality in adolescent health from Denmark, and most of these studies show persistent social inequality [7, 10, 18, 20, 25, 27, 29, 40, 44]. This new study included five indicators of physical health, five on mental health, and five on health behaviours. This study is challenged by two competing hypotheses: First, that the political ambition to reduce social inequality in health in Denmark has been successful. Second, that macroeconomic conditions have resulted in increasing social inequality in health. In general, social inequality in health increases with increasing income disparities in the society [34, 47, 48] and there has been a substantial and continuous upward trend in income inequality in Denmark during the past 30 years with an increase in the Gini-coefficient from 22 in 1991 to 30 in 2022 [49].

## Methods

### Study Design and Study Population

Data stem from the Danish arm of the international Health Behaviour in School-aged Children (HBSC) study, which collected questionnaire data among nationally representative samples of 11-, 13- and 15-year-olds about health and health behaviours [50]. The study design was cross-sectional, and data collections was repeated every 4 years following a standard protocol for sampling, measurement, and data collection. This enabled comparison of data across survey waves. Students completed the questionnaire during a school class. This study used data from nine HBSC surveys in Denmark in 1991, 1994, 1998, 2002, 2006, 2010, 2014, 2018 and 2022.

Across all waves of data collection, participants were recruited from random samples of schools, a new sample in each wave, drawn from complete lists of public and private schools in Denmark. In each school we invited all students in the fifth, seventh and ninth grade (corresponding to the age groups 11, 13 and 15) to complete the internationally standardized HBSC questionnaire in the classroom [50]. Student participation rate across all nine waves was 84.9%, n = 41,143, ranging from 90.2% in 1991 to 70.1% in 2022. School participation rate across survey waves was 37.4%, with a decline from 82.6% in 1991 to 16.0% in 2022. The most common reason for declining participation was recent participation in similar surveys. The low participation rate in 2022 was also related to schools needing to prioritize issues related to COVID-19.

### Outcome Measures

Table 1 displays the applied 15 indicators of health and health behaviours, the survey years in which they were included, their cutoff points, and validity. There were five indicators of physical health: 1) Overweight and 2) underweight based on self-reported height and weight classified by the method recommended by Cole and Lobstein [51]. 3) Headache more than once a week, 4) stomachache more than once a week, and 5) backpain more than once a week. The questions on pain originate from the HBSC Multiple Health Complaints Measure [53–56], and we used them as separate measures because pain is not only psychosomatic: these three kinds of pain may each reflect specific somatic health problems. We used the cut-off point more than once a week to separate students with severe burden of pain.

**TABLE 1:** Measurement of health indicators (Denmark, 1991–2022).

Measurement and item formulation		Response categories and cutoff points	Reliability and validity
1. Overweight (1998–2022)	Self-reported weight and height measured by the items: “How much do you weigh without clothes?” and “How tall are you without shoes?”^a^ Calculation of BMI (kg/m^2^)	Internationally standardized age- and sex-specific cutoff points [51] to categorize weight status into overweight and obese combined, normal weight, and underweight (thinness grade 2–3)	The difference in BMI calculated from self-reported and objective data are modest, on average by 0.8 kg for boys and 1.8 kg for girls [52]
2. Underweight (1998–2022)			
3. Headache more than once a week (1998–2022)	“In the last 6 months, how often have you had headache?”^a^	Items from the HBSC Multiple Health Complaints Measure [53]. Cutoff points as in reference [11] Responses dichotomized into more than once a week (“about every day” and “more than once a week”) vs. less often (“about every week,” “about every month,” and “rarely or never”)	Studies suggested that this measure is reliable assessed by consistent response patterns and valid assessed by qualitative interviews [53–56]
4. Stomachache more than once a week (1998–2022)	“In the last 6 months, how often have you had stomach-ache?”^a^		
5. Backpain more than once a week (1998–2022)	“In the last 6 months, how often have you had backpain?”^a^		
6. Daily emotional symptoms (1998–2022)	Three items from the HBSC Multiple Health Complaints Measure [53],^a^ “In the last 6 months, how often have you been … • feeling low • Irritability or • bad temper • feeling nervous	Responses dichotomized into daily (“about every day”) vs. less often (“more than once a week,” “about every week,” “about every month,” and “rarely or never”) The index separated students who answered “about every day” to at least one of these items [57]	Studies suggested that this measure is reliable assessed by consistent response patterns and valid assessed by qualitative interviews [53–56]
7. Difficulties falling asleep daily (1998–2022)	Item from the HBSC Multiple Health Complaints Measure [53].^a^“In the last 6 months, how often have you had the following: … difficulties in getting to sleep?”	Responses dichotomized into daily (“about every day”) vs. less frequent (“more than once a week,” “about every week,” “about every month,” and “rarely or never”)	
8. Loneliness often/very often (1991–1998, 2010–2022)	National item, not part of the HBSC protocol “Do you feel lonely?”	Responses dichotomised in accordance with prior studies (18.57) into lonely (“Yes very often” + “yes often”) vs. not lonely (“sometimes” + “never”)	The measurement was valid assessed by qualitative interviews [58] and correspondence with other loneliness measures [59, 60]
9. Low life satisfaction (2006–2022)	The Cantril Ladder^a^ [61] presents a ladder of 11 steps from 0 to 10 where 10 indicates the “best possible life” and 0 “the worst possible life” for you and asks: “Where on the ladder do you feel you stand at the moment?”	Cutoff points as in reference [20]: Cutoff point of 0–5 versus 6–10 to categorise low versus high score	This measure is reliable and valid for use with adolescents [61]. Responses were significantly associated with self-perception, psychological wellbeing, parent relations, mood and emotions among adolescents and therefore appears to be a useful indicator of adolescents’ life satisfaction [62]
10. Poor self-rated health (1991, 2002–2022)	1991: “What do you think about your health at present?” 2002–2022: “Would you say your health is … excellent, good, fair, poor?”^a^	Cutoff points as in reference [21]: Responses dichotomised into poor (poor, fair) and good (good, excellent)	Self-rated health is valid for disparities research in large, population-based surveys of adolescents [63]
11. Smoking, 15-year-olds (1998–2022)	“How often do you smoke?”^a^	Responses dichotomised into “every day” +“at least once a week, but not every day” vs. “less than once a week,” “I do not smoke”	Self-reported smoking among adolescents has acceptable agreement with objective measures, e.g., salivary cotinine [64, 65]
12. Drunkenness, 15-year-olds (1998–2022)	“Have you ever had so much alcohol that you were really drunk?”^a^	Cutoff points as in reference [45]: Responses dichotomized into high (”4–10 times” and “more than 10 times”) vs less. [33]	Studies suggest that adolescents’ information about their alcohol use is valid and reliable [66, 67]
13. Physical inactivity (1998–2022)	“OUTSIDE SCHOOL HOURS: How many hours a week do you usually exercise in your free time so much that you get out of breath or sweat?”^a^	Cutoff points as in reference [44]: We dichotomised the responses into “none” vs. “about half an hour” + “about 1 h” + “about 2–3 h” + “about 4–6 h” + “7 h or more”	This measure showed good reliability and a fair validity in the sense that adolescents who reported 0 h of vigorous physical activity also have low aerobic fitness [68, 69]. Toftager et al. showed that high level of self-reported vigorous physical activity corresponded with device-based measures [70]
14. Low vegetable intake (2002–2022)	“How many days a week do you usually eat vegetables?”^a^ Cutoff points as in reference [27]	Responses dichotomized into low (“never” + “less than once a week”) vs. more often (“once a week,” “2-4 days a week,” “5-6 days a week,” “once a day every day” and “every day more than once”)	A validation study reported that this measure was reliable as assessed by test-retest agreement and valid as assessed by comparison with a seven-day food diary [71]
15. Infrequent toothbrushing (1998–2022)	“How often do you brush your teeth?”^a^	Responses dichotomised according to official recommendations into frequent (“more than once a day”) vs infrequent (“once a day” + “at least once a week but not daily” + “less than once a week” + “never”)	Children’s self-reported toothbrushing habits was highly correlated with their clinically measured oral health [72]

^a^ Item from the internationally standardized HBSC questionnaire.

There were five indicators of mental health: 6) Daily emotional symptoms, an index based on three items from the HBSC Multiple Health Complaints Measure [56]. These items were so strongly intercorrelated that we suspected they measured the same aspects of emotional mental health. 7) Difficulties falling asleep every day measured by an item from the HBSC Multiple Health Complaints Measure [56]. 8) Loneliness measured by one validated item from the Danish HBSC questionnaire [58–60]; 9) low life satisfaction measured by the validated Cantril ladder [61, 62]; and 10) poor self-rated health, which is considered a valid measure encompassing aspects of both physical and mental health conditions [63]. We chose to categorize self-rated health as a mental health variable.

Finally, the study included five health compromising behaviours: 11) Smoking, only among 15-year-olds, measured by one item; 12) drunkenness, only among 15-year-olds, measured by one item. These two measurements were reported as valid at the population level [64, 66, 67]. 13) Physical inactivity measured by one item on vigorous physical activity which validly can identify a very low activity level [68–70]; 14) low vegetable intake measured by one item with acceptable validity [71], and 15) infrequent toothbrushing measured by one item with acceptable validity [72].

All outcome measures except loneliness used questions from the internationally standardized HBSC questionnaire [50]. Nine of the 15 health indicators were included in all nine survey years, and the remaining six indicators were included in most but not all survey years (Table 1). The formulation of the items and response categories in the questionnaire were similar across survey years except for a small deviation in the item about self-rated health (Table 1). Choice of cutoff points (see Table 1) was justified by two considerations, first to reflect a serious threat to adolescent health, and second to ensure enough students in the unfavourable category. Some of our cutoff points deviated from the practice within the HBSC project [50].

### Socioeconomic Measure

We measured the students’ socioeconomic status by their parents’ occupational social class (OSC). The students answered the following questions: “Does your father/mother have a job?”, “If no, why does he/she not have a job?”, “If yes, please write in what place he/she works (for example,: hospital, bank, restaurant)” and “Please write down exactly what job he/she does there (for example: teacher, bus driver).” The research group coded the responses in accordance with the Danish Occupational Social Class measure [73], ranging from I (high) to V (low) and VI for economically inactive parents who received unemployment benefits, disability pension or other kinds of transfer income, similarly based on students’ responses. The questions and coding of occupations were identical across surveys. Job titles change over time, but the coding procedure was unaffected by such changes, because we assessed occupations by two universal characteristics: 1) required educational qualifications and 2) control (over capital or people) connected with the occupation. Each participant was categorized by the highest-ranking parent into three levels of OSC: High (I-II, e.g., professionals and managerial positions, large-scale business owners), middle (III-IV, e.g., technical and administrative staff, small-scale business owners, skilled workers), and low (V, unskilled workers and VI, economically inactive). Students with insufficient information about OSC (n = 5720, 13.9% of the participants) were excluded from the analyses. The study included sex and age group as covariates.

### Statistical Procedures

This study included students with complete information about sex, age group, and the family’s OSC, n = 35,423. The analyses involved assessment of absolute social inequality estimated by prevalence differences between low and high OSC groups and relative social inequality estimated by logistic regression analyses.

We used SAS version 9.4 for the analyses. The *first step* was contingency tables to describe the prevalence of each health indicator by survey year and OSC. The *second step* was calculation of prevalence differences between low and high OSC and chi^2^-test of statistical significance of the differences between low and high OSC groups. The *third* step, time trends: We assessed time trends within each OSC group by the Cochran-Armitage test. This test aims to assess an association between a variable with two categories (here: each health indicator, one at the time) and an ordinal variable (here: survey year). The *fourth step* was logistic regression analyses to examine the sex- and age group adjusted association between the outcome variables (each health indicator, one at the time) and OSC group in each survey year. We report the results as odds ratios (OR) with 95% confidence interval (95% CI).

*Finally*, we investigated whether the social inequality changed during the study period. We conducted the logistic regression analyses for the entire period (all survey years) with inclusion of an interaction term (year*OSC) to assess if the effect of OSC on the health indicators was modified by year. Statistical interaction was reported by *p*-values (p_int_) to show whether this modification was statistically significant. The logistic regression analyses accounted for the applied cluster sampling by means of multilevel modelling (PROC GLIMMIX in SAS).

## Results

Table 2 shows the characteristics of the study population. There was an equal share of boys and girls, and an almost equal share of students in the three age groups. The OSC distribution changed over the study period. The high OSC group constituted 28.2% of the students in 1991, increasing to 52.0% in 2022. The low OSC group constituted 20.0% of the students in 1991, decreasing to 10.2% in 2022.

**TABLE 2:** School and student response rate and participants by sex, age group, and occupational social class (OSC) (Denmark, 1991–2022).

	1991	1994	1998	2002	2006	2010	2014	2018	2022	Total
Invited schools, n	23	50	64	78	100	137	168	200	588	1,408
Participating schools, n	19	45	55	68	80	73	48	45	94	527
School participation rate %	82.6	90.0	85.9	87.2	80.0	53.3	28.6	22.5	16.0	37.4
Student response rate, %	90.2	89.5	89.9	89.3	88.8	86.3	85.7	84.8	70.1	84.9
Students in the data file	1,860	4,046	5,205	4,824	6,269	4,922	4,534	3,660	5,823	41,143
Study population^a^	1,696	3,683	4,810	4,306	5,041	4,171	3,946	3,015	4,749	35,423
Pct. girls	49.9	50.7	50.4	52.0	51.5	51.0	52.1	51.4	51.7	51.3
Pct. 11-year-olds Pct. 13-year-olds Pct. 15year-olds	30.1 34.7 35.3	30.7 34.6 34.7	33.6 35.5 30.9	35.4 33.1 31.4	36.3 36.0 27.7	35.5 34.5 30.0	30.5 35.4 34.1	39.2 34.4 27.4	33.9 36.7 29.4	34.1 35.1 30.8
Pct. high OSC Pct. medium OSC Pct. low OSC	28.2 51.8 20.0	33.0 48.6 18.4	27.9 49.6 22.5	24.7 54.3 21.0	27.6 49.6 22.8	38.7 42.2 19.2	42.2 41.5 16.4	42.8 44.7 12.4	52.0 37.8 10.2	36.4 46.4 18.2

^a^ Students with information about occupational social class.

**FIGURE 1:**
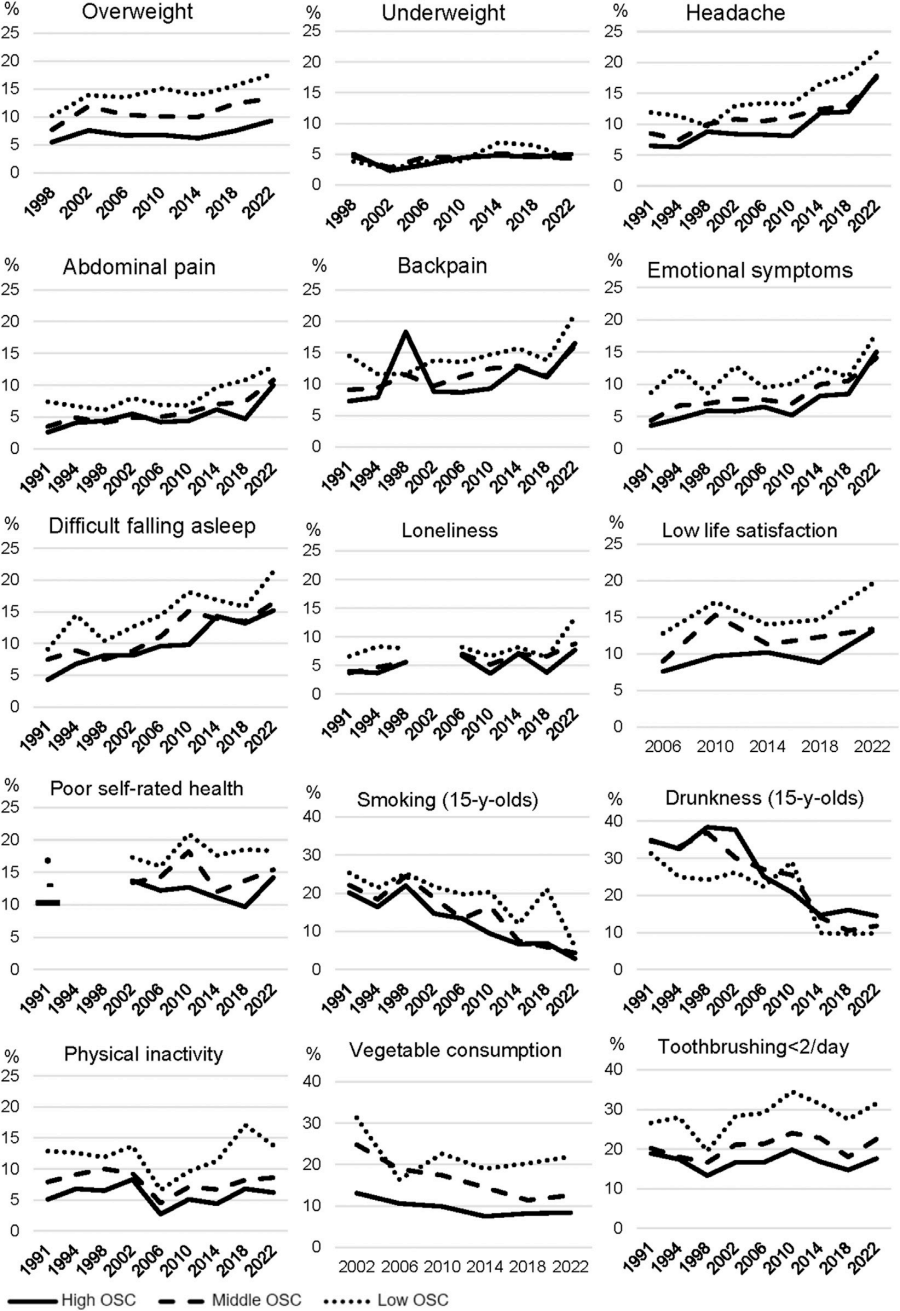
Prevalence of 15 health indicators by survey year and occupational social class (OSC) (Denmark, 1991–2022).

Figure 1 shows the prevalence (pct.) of each health indicator by OSC in each survey year from 1991 to 2022. There were fluctuations, but almost consistently across survey years the prevalence of health problems was higher in low than high OSC. There were two exceptions: underweight was not associated with OSC, and drunkenness was more prevalent in high than low OSC in most survey waves.

Tables 3–5 shows the prevalence of each health indicator in each year and in each OSC group. The columns “Prevalence Difference” shows the difference between low and high OSC group including a chi^2^-test for statistical difference. The lines “Time trend” shows the trend over time, tested by the Cochran-Armitage test.

**TABLE 3:** Physical health indicators by occupational social class; expressed as absolute and relative social inequality (Denmark, 1991–2022).

	Survey year	Absolute social inequality described by prevalence and prevalence difference (%)				Relative social inequality described by sex- and age adjusted OR (95% CI)		
		Occupational social class (OSC)				Occupational social class (OSC)		
		High	Middle	Low	Prev. Diff^a^	High	Middle	Low
Overweight	1998	5.5	7.7	10.2	4.7***	1	**1.43 (1.06–1.92)**	**2.06 (1.48–2.86)**
	2002	7.6	11.9	13.9	6.3***	1	**1.69 (1.29–2.22)**	**1.99 (1.45–2.72)**
	2006	6.7	10.4	13.5	6.8***	1	**1.61 (1.23–2.10)**	**2.20 (1.64–2.94)**
	2010	6.8	10.1	15.1	8.3***	1	**1.55 (1.18–2.03)**	**2.47 (1.82–3.36)**
	2014	6.2	10.0	13.9	7.7***	1	**1.72 (1.32–2.24)**	**2.56 (1.87–3.50)**
	2018	7.5	12.4	15.6	8.1***	1	**1.77 (1.34–2.33)**	**1.24 (1.66–2.48)**
	2022	9.3	13.3	17.6	8.3***	1	**1.50 (1.23–1.83)**	**2.11 (1.58–2.80)**
	Years (1998–2022)^b^	7.3	10.7	13.7	6.4***	1	**1.60 (1.46–1.77)**	**2.22 (1.95–2.49)**
	Time trend^c^	Up**	Up***	Up**				p_int_ = 0.9618^d^
Underweight	1998	5.0	4.6	3.8	−1.2	1	0.91 (0.65–1.27)	0.69 (0.49–1.06)
	2002	2.3	2.8	2.6	0.3	1	0.93 (0.59–1.48)	0.86 (0.48–1.54)
	2006	3.3	4.6	3.8	0.5	1	1.41 (0.97–2.04)	1.13 (0.72–1.79)
	2010	4.4	4.4	3.9	−0.5	1	0.97 (0.68–1.39)	0.84 (0.52–1.36)
	2014	4.8	5.1	6.9	2.1	1	0.99 (0.72–1.38)	1.27 (0.85–1.89)
	2018	4.5	4.8	6.4	1.9	1	1.05 (0.72–1.54)	1.39 (0.82–2.35)
	2022	5.0	4.2	4.2	−0.8	1	0.84 (0.62–1.13)	0.80 (0.48–1.33)
	Years (1998–2022)^b^	5.0	4.6	3.8	−1.2	1	1.00 (0.87–1.14)	0.95 (0.80–1.13)
	Time trend^c^	Stable	Stable	Up*				p_int_ = 0.5026^d^
Headache	1991	6.5	8.5	11.9	5.4**	1	1.38 (0.89–2.14)	**1.97 (1.20–3.26)**
	1994	6.3	7.5	11.3	5.0***	1	1.18 (0.88–1.59)	**1.82 (1.30–2.55)**
	1998	8.8	10.0	9.7	0.9	1	1.16 (0.91–1.46)	1.09 (0.82–1.44)
	2002	8.4	10.8	13.0	4.6**	1	1.26 (0.98–1.63)	**1.58 (1.17–2.12)**
	2006	8.3	10.5	13.4	5.1***	1	1.27 (1.01–1.61)	**1.69 (1.30–2.18)**
	2010	8.1	11.2	13.3	5.2***	1	**1.42 (1.12–1.79)**	**1.74 (1.32–2.29)**
	2014	11.8	12.4	16.5	4.7**	1	1.00 (0.81–1.24)	**1.41 (1.09–1.84)**
	2018	12.0	13.0	17.9	5.9**	1	1.09 (0.86–1.38)	**1.66 (1.21–2.29)**
	2022	17.8	17.5	21.6	3.8	1	1.00 (0.85–1.18)	**1.35 (1.05–1.73)**
	Years (1991–2022)^b^	10.8	11.3	13.6	2.8***	1	**1.14 (1.06–1.23)**	**1.49 (1.35–1.63)**
	Time trend^c^	Up***	Up***	Up***				p_int_ = 0.1842^d^
Stomachache	1991	2.6	3.5	7.4	4.8**	1	1.43 (0.72–2.83)	**2.85 (1.39–5.83)**
	1994	4.1	4.9	6.7	2.6*	1	1.15 (0.80–1,65)	**1.53 (1.00–2.34)**
	1998	4.4	4.1	6.1	1.7	1	0.95 (0.69–1.33)	1.31 (0.91–1.90)
	2002	5.5	4.9	8.0	2.5*	1	0.82 (0.59–1.14)	1.38 (0.96–1.99)
	2006	4.2	5.0	6.9	2.7*	1	1.15 (0.83–1.58)	**1.65 (1.16–2.34)**
	2010	4.4	5.7	6.8	2.4*	1	1.29 (0.94–1.77)	**1.54 (1.06–2.22)**
	2014	6.2	7.0	9.7	3.5*	1	1.06 (0.80–1.41)	**1.50 (1.07–2.11)**
	2018	4.7	7.4	10.8	6.1***	1	**1.59 (1.14–2.22)**	**2.44 (1.60–3.72)**
	2022	10.0	10.8	12.9	2.9	1	1.12 (0.91–1.37)	**1.35 (1.00–1.84)**
	Years (1991–2022)^b^	5.7	5.9	7.9	2.0***	1	**1.12 (1.01–1.24)**	**1.59 (1.40–1.80)**
	Time trend^c^	Up***	Up***	Up***				p_int_ = 0.3998^d^
Backpain	1991	7.3	9.1	14.5	7.2**	1	1.24 (0.81–1.90)	**2.35 (1.46–3.77)**
	1994	7.9	9.4	11.6	3.7*	1	1.21 (0.92–1.58)	**1.53 (1.11–2.22)**
	1998	8.3	11.5	11.6	3.3*	1	**1.42 (1.12–1.79)**	**1.55 (1.17–2.04)**
	2002	8.8	9.7	13.8	5.0**	1	1.11 (0.86–1.43)	**1.68 (1.26–2.23)**
	2006	8.7	11.2	13.5	4.8***	1	1.31 (1.05–1.64)	**1.66 (1.29–2.14)**
	2010	9.3	12.5	14.7	5.4***	1	**1.40 (1.12–1.75)**	**1.73 (1.33–2.25)**
	2014	12.7	12.9	15.7	3.0	1	1.00 (0.81–1.23)	1.27 (0.97–1.66)
	2018	11.1	11.3	13.8	2.7	1	1.02 (0.80–1.31)	1.33 (0.94–1.88)
	2022	16.5	16.1	21.1	4.6*	1	0.99 (0.84–1.18)	**1.45 (1.13–1.86)**
	Years (1991–2022)^b^	10.9	11.6	14.0	3.1***	1	**1.15 (1.07–1.25)**	**1.52 (1.39–1.67)**
	Time trend^c^	Up***	Up***	Up***				p_int_ = 0.2842^d^

^a^ Prevalence in low OSC minus prevalence in high OSC.

^b^ Logistic regression analyses combining all survey years adjusted for sex, age group, and survey year.

^c^ Time trends assessed by Cochran-Armitage test. Statistical significance at the 95% level: *<0.05, **<0.001, **<0.0001.

^d^ p_int_ is the *p*-value for statistical interaction between year and OSC.

Bold text indicating significant results.

**TABLE 4:** Mental health indicators by occupational social class; expressed as absolute and relative social inequality (Denmark, 1991–2022).

	Survey year	Absolute social inequality described by prevalence and prevalence difference (%)				Relative social inequality described by sex- and age adjusted OR (95% CI)		
		Occupational social class (OSC)				Occupational social class (OSC)		
		High	Middle	Low	Prev. Diff^a^	High	Middle	Low
Emotional symptoms	1991	3.6	4.4	8.7	5.1*	1	1.21 (0.67–2.17)	**2.53 (1.36–4.70)**
	1994	4.7	6.7	12.4	7.7***	1	**1.44 (1.04–2.00)**	**2.79 (1.96–3.99)**
	1998	5.9	7.0	8.6	2.7*	1	1.21 (0.92–1.60)	**1.49 (1.02–2.06)**
	2002	5.8	7.7	12.8	7.0***	1	1.29 (0.95–1.74)	**2.29 (1.65–3.18)**
	2006	6.5	7.6	9.5	3.0*	1	1.16 (0.89–1.50)	**1.49 (1.11–1.99)**
	2010	5.2	7.0	10.1	4.9***	1	**1.34 (1.01–1.80)**	**1.86 (1.43–2.73)**
	2014	9.2	10.0	12.5	3.3***	1	1.03 (0.81–1.30)	1.32 (0.98–1.77)
	2018	8.5	10.5	11.2	2.7	1	1.24 (0.95–1.62)	1.37 (0.94–2.00)
	2022	15.0	14.1	17.9	2.9	1	1.09 (0.79–1.13)	1.28 (0.98–1.67)
	Years (1991–2022)^b^	8.2	8.4	11.2	3.0***	1	**1.13 (1.04–1.23)**	**1.63 (1.47–1.81)**
	Time trend^c^	Up***	Up***	Up**				p_int_ = 0.0122^d^
Difficulties falling asleep	1991	4.3	7.5	9.1	4.8*	1	**1.79 (1.07–3.00)**	**2.15 (1.20–3.87)**
	1994	6.8	8.9	14.5	7.7***	1	1.30 (0.98–1.72)	**2.22 (1.62–3.05)**
	1998	8.1	7.5	10.4	2.3	1	0.92 (0.72–1,19)	1.22 (0.92–1.62)
	2002	8.1	8.8	12.6	4.5***	1	1.07 (0.82–1.40)	**1.58 (1.17–2.14)**
	2006	9.6	11.1	14.4	4.8**	1	1.15 (0.93–1.44)	**1.55 (1.21–1.98)**
	2010	9.8	15.1	18.1	8.3***	1	**1.63 (1.32–2.01)**	**2.00 (1.56–2.56)**
	2014	14.3	13.9	16.9	2.6	1	0.95 (0.77–1.16)	1.17 (0.90–1.51)
	2018	13.2	13.5	15.8	2.6	1	1.02 (0.81–1.27)	1.21 (0.88–1.68)
	2022	15.2	16.4	21.3	6.1*	1	1.09 (0.93–1.30)	**1.49 (1.16–1.91)**
	Years (1991–2022)^b^	11.0	11.3	14.5	3.5***	1	**1.33 (1.05–1.22)**	**1.53 (1.39–1.67)**
	Time trend^c^	Up***	Up***	Up***				p_int_ = 0.0081^d^
Loneliness	1991	4.0	3.7	6.6	2.6	1	0.93 (0.52–1.65)	1.65 (0.88–3.12)
	1994	3.7	4.7	8.3	4.6***	1	1.26 (0.87–1.83)	**2.26 (1.51–3.39)**
	1998	5.6	5.6	8.0	2.4*	1	1.00 (0.75–1.35)	**1.47 (1.06–2.03)**
	2010	3.6	5.2	6.6	3.0*	1	1.47 (0.96–2.23)	**1.86 (1.15–3.02)**
	2014	7.1	7.0	8.2	1.1	1	0.98 (0.73–1.25)	1.17 (0.83–1.65)
	2018	3.8	6.6	6.4	2.6*	1	**1.75 (1.22–2.50)**	**1.76 (1.06–2.93)**
	2022	7.7	8.8	13.3	5.6***	1	1.18 (0.94–1.48)	**1.94 (1.41–2.66)**
	Years (1991–1998, 2010–2022)^b^	5.8	6.3	8.2	3.4***	1	**1.14 (1.02–1.27)**	**1.57 (1.37–1.79)**
	Time trend^c^	Up***	Up***	Up*				p_int_ = 0.0401^d^
Low life satisfaction	2006	7.6	9.0	12.8	5.2***	1	1.18 (0.92–1.50)	**1.78 (1.37–2.33)**
	2010	9.7	15.3	17.1	7.4***	1	**1.65 (1.33–2.04)**	**1.88 (1.47–2.42)**
	2014	10.2	11.3	13.7	2.5*	1	1.07 (0.86–1.34)	**1.36 (1.03–1.80)**
	2018	8.8	12.3	14.7	5.9**	1	**1.44 (1.12–1.86)**	**1.80 (1.26–2.55)**
	2022	13.1	13.4	19.6	6.5**	1	1.05 (0.87–1.26)	**1.73 (1.33–2.24)**
	Years (2006–2022)^b^	10.4	11.9	15.2	4.8***	1	**1.21 (1.02–1.33)**	**1.66 (1.49–1.86)**
	Time trend^c^	Up***	Up**	Up*				p_int_ = 0.0762^d^
Poor self-rated health	1991	10.6	13.1	16.6	6.0*	1	1.28 (0.90–1.83)	**1.79 (1.19–2.71)**
	2002	13.8	13.2	17.5	3.7*	1	0.97 (0.78–1.20)	**1.34 (1.04–1.71)**
	2006	12.3	14.1	15.7	3.4*	1	1.18 (0.97–1.43)	**1.35 (1.08–1.70)**
	2010	12.8	18.2	21.2	8.4***	1	**1.52 (1.25–1.85)**	**1.84 (1.46–2.31)**
	2014	11.1	12.2	18.2	7.1***	1	1.08 (0.87–1.34)	**1.74 (1.34–2.25)**
	2018	9.8	13.5	18.6	8.8***	1	**1.47 (1.15–1.88)**	**2.13 (1.54–2.84)**
	2022	14.2	15.2	18.5	4.3*	1	1.12 (0.94–1.33)	**1.42 (1.09–1.84)**
	Years (1991, 2002–2022)^b^	12.4	14.3	17.9	5.5***	1	**1.19 (1.14–1.29)**	**1.54 (1.40–1.69)**
	Time trend^c^	Stable	Stable	Stable				p_int_ = 0.0176^d^

^a^ Prevalence in low OSC minus prevalence in high OSC.

^b^ Logistic regression analyses combining all survey years adjusted for sex, age group, and survey year.

^c^ Time trends assessed by Cochran-Armitage test. Statistical significance at the 95% level: *<0.05, **<0.001, **<0.0001.

^d^ p_int_ is the *p*-value for statistical interaction between year and OSC.

Bold text indicating significant results.

**TABLE 5:** Health behaviours by occupational social class; expressed as absolute and relative social inequality (Denmark, 1991–2022).

	Survey year	Absolute social inequality described by prevalence and prevalence difference (%)				Relative social inequality described by sex- and age adjusted OR (95% CI)		
		Occupational social class (OSC)				Occupational social class (OSC)		
		High	Middle	Low	Prev. Diff^a^	High	Middle	Low
Smoking weekly (15-year-olds)	1991	20.1	22.1	25.3	5.2	1	1.14 (0.71–1.82)	1.36 (0.74–2.48)
	1994	16.4	18.4	21.4	5.0	1	1.22 (0.78–1.82)	0.93 (0.51–1.73)
	1998	22.0	24.5	25.2	3.2	1	1.13 (0.86–1.49)	1.18 (0.82–1.70)
	2002	14.7	18.8	21.8	7.1*	1	1.32 (0.94–1.86)	**1.60 (1.06–2.41)**
	2006	13.4	13.3	19.7	6.3*	1	1.01 (0.70–1.45)	**1.62 (1.07–2.44)**
	2010	9.4	16.5	20.3	10.9***	1	**2.14 (1.13–4.06)**	**2.72 (1.31–5.65)**
	2014	6.7	7.5	11.9	5.1*	1	1.14 (0.73–1.79)	**1.89 (1.10–3.26)**
	2018	6.9	5.9	21.1	14.2***	1	0.63 (0.17–2.97)	**3.87 (1.18–12.7)**
	2022	2.9	4.4	5.9	3.0	1	1.56 (0.86–2.83)	2.17 (0.90–5.22)
	Years (1991–2022)^b^	11.1	15.3	19.6	8.5***	1	**1.22 (1.07–1.39)**	**1.63 (1.39–1.91)**
	Time trend^c^	Down***	Down***	Down***				p_int_ = 0.0764^d^
Drunkenness (15-year-olds)	1991	43.4	46.3	38.1	−5.3	1	1.11 (0.76–1.63)	0.79 (0.47–1.63)
	1994	45.3	41.3	33.3	−12.0*	1	0,86 (0.67–1.10)	**0.61 (0.42–0.86)**
	1998	50.2	48.2	39.3	−10.9*	1	0.84 (0.74–1.18)	**0.65 (0.47–0.89)**
	2002	47.5	44.1	39.3	−8.2	1	0.89 (0.69–1.14)	0.73 (0.53–1.00)
	2006	37.5	36.3	32.9	−6.6	1	0.96 (0.74–1.25)	0.83 (0.60–1.14)
	2010	36.8	40.8	38.2	2.2	1	1.19 (0.92–1.53)	1.06 (0.76–1.49)
	2014	23.1	21.5	14.4	−8.7*	1	0.93 (0.70–1.23)	**0.58 (0.37–0.90)**
	2018	27.5	21.9	25.3	−2.2	1	0.77 (0.54–1.08)	0–91 (0.51–1.61)
	2022	25.0	26.5	18.6	−6.1	1	1.08 (0.84–1.40)	0.68 (0.42–1.11)
	Years (1991–2022)^b^	35.5	37.3	32.4	−1.1**	1	0.96 (0.88–1.05)	**0.75 (0.66–0.84)**
	Time trend^c^	Down***	Down***	Down***				p_int_ = 0.5245^d^
Physical inactivity	1991	5.1	7.9	12.9	7.8***	1	1.59 (0.99–2.58)	**2.78 (1.65–4.68)**
	1994	6.8	9.1	12.6	5.8***	1	**1.36 (1.03–1.80)**	**1.98 (1.44–2.73)**
	1998	6.5	10.0	11.9	5.4***	1	**1.60 (1.23–2.06)**	**2.06 (1.54–2.74)**
	2002	8.3	9.3	13.7	5.4***	1	1.14 (0.88–1.48)	**1.80 (1.34–2.41)**
	2006	2.7	4.5	6.6	3.9***	1	**1.70 (1.16–2.48)**	**2.61 (1.74–3.90)**
	2010	5.1	7.1	9.6	4.5***	1	**1.44 (1.07–1.92)**	**2.09 (1.50–2.89)**
	2014	4.4	6.7	11.3	6.9***	1	**1.56 (1.14–2.13)**	**2.82 (1.98–4.01)**
	2018	6.8	8.2	17.1	10.3***	1	**1.23 (0.91–1.66)**	**2.83 (1.97–4.07)**
	2022	6.2	8.6	13.8	7.6***	1	**1.44 (1.13–1.93)**	**2.51 (1.83–3.46)**
	Years (1991–2022)^b^	5.7	7.9	11.4	5.7***	1	**1.43 (1.29–1.57)**	**2.34 (2.00–2.50)**
	Time trend^c^	Stable	Down*	Stable				p_int_ = 0.4134^d^
Low vegetable intake	2002	13.1	24.8	31.3	18.2***	1	**2.24 (1.83–2.75)**	**3.12 (2.48–3.92)**
	2006	10.6	18.9	26.4	15.8***	1	**2.02 (1.65–2.49)**	**3.09 (2.49–3.84)**
	2010	9.9	17.4	22.6	12.7***	1	**1.94 (1.58–2.39)**	**2.75 (2.17–3.48)**
	2014	7.5	14.5	18.9	11.4***	1	**2.17 (1.72–2.74)**	**3.03 (2.30–3.98)**
	2018	8.2	11.4	20.3	12.1***	1	**1.45 (1.12–1.89)**	**2.83 (2.05–3.92)**
	2022	8.4	12.6	21.9	13.5***	1	**1.57 (1.28–1.92)**	**3.05 (2.35–3.96)**
	Years (2002–2022)^b^	9.3	17.0	24.6	15.3***	1	**1.90 (1.74–2.07)**	**2.91 (2.63–3.22)**
	Time trend^c^	Down***	Down***	Down***				p_int_ = 0.1540^d^
Infrequent toothbrushing	1991	18.9	20.2	26.6	7.7*	1	1.08 (0.82–1.45)	**1.58 (1.13–2.22)**
	1994	17.4	18.0	28.0	10.6^***^	1	1.08 (0.89–1.31)	**1.97 (1.57–2.49)**
	1998	13.3	16.6	19.5	6.2***	1	**1.29 (1.07–1.57)**	**1.59 (1.28–1.99)**
	2002	16.6	21.1	28.3	11.7***	1	**1.38 (1.14–1.67)**	**2.04 (1.64–2.55)**
	2006	16.6	21.3	29.1	12.5***	1	**1.40 (1.18–1.66)**	**2.13 (1.76–2.57)**
	2010	19.8	24.0	34.5	14.7***	1	**1.29 (1.09–1.52)**	**2.17 (1.79–2.64)**
	2014	16.8	22.8	31.4	14.6***	1	**1.49 (1.25–1.78)**	**2.32 (1.88–2.87)**
	2018	14.7	18.0	27.5	12.8***	1	**1.27 (1.03–1.57)**	**2.21 (1.68–2.92)**
	2022	17.6	22.5	31.5	13.9***	1	**1.36 (1.17–1.58)**	**2.06 (1.65–2.57)**
	Years (1991–2022)^b^	16.9	20.5	28.0	11.1***	1	**1.32 (1.24–1.40)**	**2.02 (1.87–2.17)**
	Time trend^c^	Stable	Up***	Up***				p_int_ = 0.3338^d^

^a^ Prevalence in low OSC minus prevalence in high OSC.

^b^ Logistic regression analyses combining all survey years adjusted for sex, age group, and survey year.

^c^ Time trends assessed by Cochran-Armitage test. Statistical significance at the 95% level: *<0.05, **<0.001, **<0.0001.

^d^ p_int_ is the *p*-value for statistical interaction between year and OSC.

Bold text indicating significant results.

### Overall Development in Health

There was a significant deterioration in physical health measured by overweight, headache, stomachache, and backpain in all OSC groups and for underweight in the low OSC group (Table 3). There was also a significant deterioration in mental health measured by daily emotional symptoms, loneliness, difficulties falling asleep, and poor life satisfaction in all OSC groups (Table 4). There was improvement in smoking and drunkenness behaviour and vegetable intake in all OSC groups and mixed developments in physical inactivity and infrequent toothbrushing (Table 5).

### Social Inequality

There was an *absolute social inequality* (prevalence difference) in 13 of 15 health indicators in most survey years (Tables 3–5). There were two exceptions: Underweight was not associated with OSC and drunkenness was more prevalent in high than low OSC. Assessed by OR estimates, there was a *relative social inequality* in 13 of 15 health indicators in most survey years, again with the same two exceptions: Underweight was not associated with OSC and drunkenness was more common in high than low OSC. Two examples of the social inequality in health were the OR (95% CI) for overweight in low socioeconomic groups which was 2.22 (1.95–2.49) and for low vegetable intake, 2.91 (2.63–3.22).

### Trends in Social Inequality

The absolute social inequality (prevalence difference) fluctuated across the study period without any consistent increasing or decreasing patterns (Tables 3–5). There was one exception: the prevalence difference for overweight increased from 4.7 in 1998 to 8.3 in 2022. The relative social inequality assessed by OR-estimates also fluctuated across survey years without any consistent patterns. Tests for statistical interaction suggested that survey year modified the social inequality in daily emotional symptoms (p_int_ = 0.0122), in difficulties falling asleep (p_int_ = 0.0081), in loneliness (p_int_ = 0.0401), and in poor self-rated health (p_int_ = 0.0176). The broad confidence intervals in the analyses of relative social inequality did not allow any conclusion about increasing or diminishing social inequality. Further, eyeballing of the trendlines in Figure 1 suggest that there might be a slight reduction in social inequality for these four health indicators but that the significant p_int_-values could as well reflect more random variations in trendlines.

## Discussion

### Main Findings

This is the first study of secular trends in social inequality in adolescents’ health which covers a period of more than three decades and a broad selection of health indicators. There were two main findings. First, there was persistent social inequality – meaning more problems in lower OSC-groups - in 13 of 15 health indicators from 1991 to 2022. The social inequality appeared in absolute terms (prevalence differences) as well as in relative terms (odds ratios). Drunkenness among 15-year-olds was persistently more prevalent in high than low OSC and underweight was persistently not associated with OSC. The observation of trends in social inequality in adolescents’ health aligns with many other studies covering overweight [6–8], pain [10, 12], mental health problems [12, 15, 17] and unhealthy behaviours [6, 24, 27].

Second, the magnitude of social inequality in health fluctuated from one survey year to the next but did not change in any systematic way over the past three decades. Test for statistical interaction suggested a slight reduction in social inequality in four mental health indicators (daily emotional symptoms, difficulties falling asleep, loneliness, poor self-rated health). This finding did not consistently support any of the two competing hypotheses: The health policy goal to reduce social inequality in health was not achieved, and the increasing income inequality in the past three decades did not result in increasing social inequality in health. Several studies show widening social inequality in physical inactivity, overweight, and smoking [6, 31, 34, 35, 38, 41] as well as slightly widening social inequality in pain, psychological distress, loneliness, difficulties in falling asleep, and poor self-rated health [1, 12, 13, 22, 39, 74]. From a health policy point of view, it is disappointing that the desired reduction of social inequality was unsuccessful. The efforts may have been insufficient, or other developments in the society may have facilitated increasing social inequality. It is a challenge to explain the almost universal and persistent pattern of social inequality in adolescent health, but the Theory of Fundamental Causes [75] provides a potential explanatory framework: According to this theory, the reason for persistent social inequality is that high socioeconomic status embodies a multitude of resources (material assets, knowledge, control, resourceful social networks, etc.) which protect health, no matter what mechanisms are at stake.

### Methodological Issues

The strength of the HBSC study is that it covers many indicators of adolescent health, covers an extended period, and that the survey rounds are methodologically comparable [50, 76]. The included health indicators are important aspects of adolescent health as they either challenge the life quality of the individuals and/or increase the risk of future disease. Several other important indicators such as hospitalization, healthcare use, chronic illness, and diagnosed mental health problems were not included in the study.

There are important limitations as well. *First*, there may be problems related to comparability of socioeconomic status over time. We used occupational social class, a generic indicator of socioeconomic status which reflects the family’s position in the occupational structure [77]. We have reasons to believe that the data – although with a high level of missing data - are valid because most students in these age groups can report their parents’ occupation with a reasonable validity [76, 78–81]. Pförtner et al. showed that OSC is an appropriate variable for studies of social inequality in adolescents’ health [82]. The occupational structure in the country changed substantially from the 1990s to 2022 and so did the OSC distribution in the population. The traditional working classes shrieked, and the upper middle classes increased in size. Therefore, widening social inequality may reflect changes in the composition of the population. We decided not to use the socioeconomic indicator, which is often used in analyses of HBSC data, the Family Affluence Scale (FAS) [83] because FAS was not available for the two first surveys in 1991 and 1994, and FAS has relatively low correlations with two generic measures of socioeconomic status, parental education and parents’ occupational status [84].

A *second* limitation may be selection bias. We excluded 5,720 students with incomplete information about parents’ OSC, 13.9% of the sample. We also excluded students with missing data on each health indicator. In most cases the proportion of missing data on health indicators was < 5% but in the analyses of overweight and underweight we missed 12.9% of the applied study population. We have no way to investigate the magnitude or direction of the potential selection bias.

### Implications

We need more insight into how socioeconomic health differences change over time. We need to know more about other health indicators; whether changes in social inequality vary by country; and whether these social inequalities are sensitive to health policy interventions. We may need other inequality methodologies. Although regression-based measures such as Slope Index of Inequality and Relative Index of Inequality are sensitive to changes in the distribution of the socioeconomic groups over time, they could still result in misleading conclusions regarding changes in the social inequality [85]. When it comes to health policy monitoring, we might consider total impact inequality measures such as Population Attributable Fraction and Index of Dissimilarity more relevant for future studies.

From a policy point of view, there is a desire to fight social inequalities in adolescent health, because they limit the full health potential of adolescents from lower socioeconomic groups. The substantial efforts in England over the past decades to reduce social inequality in child and adolescent health were disappointing [4, 86]. Mackenbach concluded that “Health inequalities can be reduced substantially only if governments have a democratic mandate to make the necessary policy changes, if demonstrably effective policies can be developed and if these policies are implemented on the scale needed to reach the overall targets.” [4]. Other scholars suggest that less radical ways to tackle social inequality in child and adolescent health may be successful. Diderichsen et al. suggest a combination of structural changes such as reducing child poverty, reducing early school drop-out, and fighting harmful health behaviours [87]. Law et al. suggest that child health professionals can contribute by ensuring that health services are accessible and equitable. They also emphasize that staff training could foster an understanding of the causes and solutions to child health inequalities [86].

### Conclusion

There was significant social inequality in 13 of 15 specific indicators of adolescent health and this pattern did not change much in the period 1991–2022. There is a need for better monitoring of social inequalities in adolescent health and for strengthened policies to improve adolescent health across socioeconomic groups.
